# Effect of a Population Health Management Intervention on Medication Therapy Problems in People With Chronic Kidney Disease: Post Hoc Analysis of the K-CHAMP Cluster-Randomized Trial

**DOI:** 10.1016/j.xkme.2025.100995

**Published:** 2025-03-18

**Authors:** Melanie R. Weltman, Zhuoheng Han, Linda-Marie U. Lavenburg, Alaa A. Alghwiri, Jonathan G. Yabes, Thomas D. Nolin, Manisha Jhamb

**Affiliations:** 1 Renal-Electrolyte Division, Department of Medicine, University of Pittsburgh School of Medicine, Pittsburgh, Pennsylvania; 2 Department of Pharmacy and Therapeutics, University of Pittsburgh School of Pharmacy, Pittsburgh, Pennsylvania; 3 Division of General Internal Medicine, Department of Medicine and Biostatistics, Center for Research on Heath Care, University of Pittsburgh, Pittsburgh, Pennsylvania

**Keywords:** Chronic kidney disease, medication discrepancy, medication therapy problem, multidisciplinary team, population health management

## Abstract

**Rationale & Objective:**

Medication therapy problems (MTPs) are therapeutic issues related to medications that may cause undesirable events. People with chronic kidney disease (CKD) are at high risk of experiencing MTPs owing to comorbid conditions and medication burden. This study characterizes MTPs in individuals enrolled in the Kidney Coordinated Health Management Partnership trial and evaluates the intervention’s effect on MTPs.

**Study Design:**

Post hoc analysis of a pragmatic, cluster-randomized trial.

**Setting & Participants:**

Individuals aged 18-85 years with an estimated glomerular filtration rate of <60 mL/min/1.73 m^2^, moderate to high risk of CKD progression, and not seeing a nephrologist enrolled from 101 primary care practices (May 2019 to November 2021).

**Intervention(s):**

Electronic health record-based multidisciplinary care including nephrology e-consult, pharmacist medication review, and patient education at baseline and every 6 months.

**Outcomes:**

MTP type and frequency of occurrence were characterized along with associated medication classes. Descriptive statistics of MTPs were conducted, and cumulative probabilities of resolution over time were estimated using the discrete-time survival method.

**Results:**

Baseline medication reviews were completed by telephone (52%) or chart review (48%) in 730 out of 754 (97%) intervention-arm participants (mean age, 74 ± 9 years and estimated glomerular filtration rate, 37 ± 8 mL/min/1.73 m^2^). Polypharmacy was evident in 63% of participants. At baseline, 78% had MTPs and 79% had medication discrepancies. The most common MTP was indication without drug therapy, associated with sodium-glucose cotransporter-2 (SGLT-2) inhibitors. The average number of MTPs per participant decreased from 2.01 at baseline to 1.28 at 6 months (36% reduction), and 1.15 at 12 months (43% reduction). Based on the discrete-time survival model, an estimated 92% of MTPs were resolved by 12 months.

**Limitations:**

Medication management was not completed for control-arm participants. No standardized tool was used to assess medication adherence. We relied on electronic health record chart review to identify MTPs in participants who could not be reached by telephone.

**Conclusions:**

MTPs and medication discrepancies are highly prevalent in nondialysis-dependent CKD. Medication management through multidisciplinary team care can optimize medication therapy in CKD.

## Plain-Language Summary

People with chronic kidney disease (CKD) are at risk of experiencing medication therapy problems (MTPs), which are issues related to medications that may cause undesirable events. In this study, we characterized the types of MTPs experienced by people with CKD in the primary care setting and evaluated the effect of a multidisciplinary intervention on MTPs. At baseline, 78% of participants had MTPs. The most common problem was not being prescribed a medication for a diagnosed condition. The average number of MTPs per participant decreased by 43% at 12 months. This study helped us to understand MTP characteristics in people with CKD and shows that multidisciplinary team care can optimize medications.

Medication management and drug stewardship are highlighted by the KDIGO (Kidney Disease: Improving Global Outcomes) 2024 Clinical Practice Guideline for The Evaluation and Management of Chronic Kidney Disease (CKD) as the key components of CKD care.^1^ Individuals with CKD exhibit high medication burden—people with stage 3-4 CKD take, on average, 6-8 medications concurrently and those with CKD stage 5 take ≥12 medications.^2^^,^^3^ Polypharmacy (≥5 concomitant medications) is present in almost 70%, and hyperpolypharmacy (≥10 concomitant medications) in 24% of people with CKD.^4^ With the emergence of new guideline-recommended medications for CKD, medication burden, and associated problems such as nonadherence, drug-drug interactions and medication errors may worsen. Alterations in pharmacokinetics and pharmacodynamics in CKD further complicate medication regimens by necessitating careful medication drug class selection and dose adjustments as the disease progresses.^5^ In addition, people with CKD are at risk of taking inappropriate medications that are associated with adverse outcomes and may necessitate deprescribing.^6^ Thus, medication management by trained clinical pharmacists is a core element of multidisciplinary team care to optimize medication therapy for individuals with CKD.

Medication therapy problems (MTPs) are “any undesirable event experienced by a patient that involves, or is suspected to involve, drug therapy, and that interferes with achieving the desired goals of therapy and requires professional judgment to resolve.”^7^ MTPs include therapeutic issues related to medication effectiveness, safety, or adherence. MTPs are associated with significant morbidity, mortality, and increased health care costs. The estimated annual cost of drug-related morbidity and mortality owing to nonoptimized medication therapy is $528.4 billion.^8^ Individuals with CKD are vulnerable to MTPs owing to multimorbidity, polypharmacy, and the need for frequent medication regimen changes.^3^ Furthermore, people with CKD have up to 40% higher mortality rate associated with exposure to contraindicated drugs or drugs requiring renal dose adjustment compared with people without CKD.^9^

MTPs are common in all stages of CKD. They are well documented in individuals with dialysis-dependent CKD; medication management services involving dedicated pharmacists have demonstrated several benefits including lower health care spending and a trend toward improved quality of life in this population.^2^^,^^10^^,^^11^ However, there is a critical knowledge gap among individuals with nondialysis-dependent CKD. Most studies either evaluate MTPs in hospital settings or post hospital discharge, or include a small sample of participants with no-dialysis-dependent CKD in a predominantly dialysis-dependent CKD cohort.^12^^,^^13^ Understanding common MTPs in the outpatient setting is important to potentially reduce adverse events, prevent hospitalizations related to suboptimal medication management,^14^, ^15^, ^16^ and increase access to novel therapies.^17^^,^^18^ This study aimed to characterize MTPs in individuals with nondialysis-dependent CKD who participated in the Kidney Coordinated Health Management Partnership (K-CHAMP) trial, which tested a multidisciplinary population health management intervention versus usual care on CKD evidence-based care delivery in the primary care setting. In addition, we evaluated changes in MTPs after multidisciplinary population health management intervention, which included medication review by a clinical pharmacist.

## Methods

### Design, Setting, and Population

This study analyzed data from the K-CHAMP trial (ClinicalTrials.gov; NCT03832595)—a pragmatic, cluster-randomized trial in individuals with moderate- to high-risk CKD (May 2019 to July 2022).^19^ K-CHAMP enrolled 1,596 participants (754 intervention and 842 usual-care control) from 101 cluster-randomized University of Pittsburgh Medical Center primary care practices. Participants were identified using an electronic health record (EHR)-based CKD registry. Eligibility criteria included age 18-85 years, estimated glomerular filtration rate (eGFR) < 60 mL/min/1.73 m^2^, high-risk CKD defined as eGFR 15-29 mL/min/1.73 m^2^, 5-year risk of kidney failure ≥ 4% using validated 4-variable kidney failure risk equation,^20^ established with an included primary care provider (PCP), and no outpatient nephrology visit in the last 12 months. People with a history of kidney transplant, receiving maintenance dialysis, or with baseline eGFR < 15 mL/min/1.73 m^2^ were excluded. The K-CHAMP primary outcome was the time from first PCP visit to ≥40% reduction in eGFR or kidney failure.^21^ The study protocol was approved by the Institutional Review Board and the Quality Improvement Committee at the University of Pittsburgh. Participants were provided an opportunity to opt-out of the study entirely, or could opt-out of pharmacist contact. In this post hoc analysis, participants who received at least 1 medication review by the study pharmacist (either via telephone or via chart review) at baseline were included, totaling 730 (97%) intervention-arm participants. Medication reviews were not completed for participants enrolled in the control arm, thus they were not included in this analysis.

### Intervention

Before the intervention period, a study guideline based on current clinical practice guidelines and standardized drug information resources (Food and Drug Administration labeling information; UpToDate and Micromedex) was created by the clinical team to address recommendations related to medication dosing, medication safety, and clinical nephrology, which included management of hypertension, diabetes mellitus, hyperkalemia, atherosclerotic cardiovascular disease risk, and immunizations. The study guideline was periodically updated to reflect new clinical practice guidelines published during the intervention period, including yearly updated American Diabetes Association Standards of Care in Diabetes and the KDIGO 2020 Clinical Practice Guideline for Diabetes Management in Chronic Kidney Disease.^22^, ^23^, ^24^, ^25^

The intervention was a multifaceted bundle of nephrology care services that included the following: (1) nephrology specialist (nephrologist or advanced practice provider)--led targeted automated e-consults, (2) pharmacist-led medication management services, and (3) nurse-led CKD education.^19^

MTPs were identified through comprehensive medication reviews (CMRs) completed by pharmacists. Per our study protocol, the medication review was completed by phone (preferably) or chart review based on patient preference and availability. For each encounter, the pharmacist made 3 attempts by phone and sent a secure message through the EHR patient portal (when available) to contact the participant. During the CMR, the pharmacist reviewed the patient’s self-reported medication regimen, including prescription and nonprescription medications, administration routine, and medication adherence. If participants could not be reached or declined CMR, a chart review of medication history, recent medication dispensing history, problem list, and relevant laboratory values was completed to identify potential MTPs. After discussion with a clinical team of nephrologists, advanced practice providers, pharmacists, and nurse educators, a comprehensive plan was developed for each participant. The pharmacists used a standardized note template to provide recommendations for medication optimization to the PCP (Box S1). To align with PCP workflow, recommendations were provided via the EHR to the participant’s PCP within 1 week before the participant’s primary care appointment. Follow-up CMRs occurred approximately every 6 months for the duration of the study period and were completed by telephone, or chart review, if the patient could not be reached by telephone. The frequency of medication review was based on Centers for Medicare & Medicaid Services Medication Therapy Management Programs, pharmacy practice standards for outpatient nephrology settings, and KDIGO guidance.^26^^,^^27^

### MTP Classification

During each pharmacist encounter, MTPs were identified and classified into the following 9 categories: indication without drug therapy, drug use without indication, suboptimal drug, dosage too low, dosage too high, adverse drug reaction, drug interaction, failure to receive drug, and inappropriate laboratory monitoring (Table 1).^3^^,^^7^^,^^11^ When CMR was completed by telephone, medication discrepancies were also identified and documented. Medication discrepancies were defined as any differences between the participant-reported medication list and the EHR-documented medication list, and were categorized as drug no longer taken, drug not recorded in EHR, drug dose changed, and different administration directions. Medication discrepancies may be undocumented intentional discrepancies (ie, prescription from another provider or appropriate over-the-counter medication use) and do not necessarily imply the occurrence of MTPs, which are characterized by harm or risk of harm to a patient. However, medication discrepancies may lead to future MTPs such as duplications of therapy, drug-drug interactions, or adverse drug events.^28^^,^^29^

**Table 1:** Top 3 Medication Classes Commonly Associated With MTP Categories at Baseline

MTP Category (N at Baseline)	Top 3 Medication Classes in Each Category (N, %)
Indication without drug therapy (348) (patient not prescribed medication for a diagnosed condition)	SGLT-2i (115, 33.0) ACEi/ARB (65, 18.7) Statin (63, 18.1)
Suboptimal drug (217) (medication of choice not being used; contraindication present)	NSAID (61, 28.1) Thiazide diuretic (24, 11.1) Biguanide (16, 7.4)
Dosage too high (154) (patient treated with too much of the correct medication)	Biguanide (37, 24.0) Histamine H2 receptor antagonist (29, 18.8) GABA analog (24, 15.6)
Dosage too low (140) (patient treated with too little of the correct medication to produce the desired response)	ACEi/ARB (60, 42.9) Statin (29, 20.7) GLP-1 receptor agonist (14, 10)
Drug use without indication (67) (use of a medication without a medically valid indication)	Proton pump inhibitor (37, 55.2) NSAID (6, 9.0) GLP-1 receptor agonist (2, 3.0)
Drug interaction (86) (drug-drug, drug-disease, or drug-food interaction)	Fibrate-statin (24, 27.9) Calcium channel blocker-statin (21, 24.4) GABA analog-opioid analgesic (4, 4.7)
Inappropriate laboratory monitoring (49) (patient not receiving appropriate laboratory tests to monitor medication therapy)	ACEi/ARB (9, 18.4) Diguanide (8, 16.3) Xanthine oxidase inhibitor (8, 16.3)
Adverse drug reaction (48) (adverse effects that are unwanted, unpleasant, or harmful)	ACEi/ARB (7, 14.6) Calcium channel blocker (6, 12.5) Thiazide diuretic (6, 12.5)
Failure to receive drug (44) patient not receiving prescribed medication(s))	Statin (8, 18.2) ACEi/ARB (6, 13.6) Loop diuretic (4, 9.1)

*Note:* MTP definitions adapted from Cardone et al^3^ and Cipolli et al.^7^

Abbreviations: ACEi, angiotensin-converting enzyme inhibitor; ARB, angiotensin II receptor blocker; GABA, gamma-aminobutyric acid; GLP-1RA, glucagon-like peptide-1 receptor agonist; MTP, medication therapy problem; NSAID, nonsteroidal anti-inflammatory drug; SGLT-2i, sodium-glucose cotransporter-2 inhibitor.

### Outcome and/or Objective

The aim of this study was to characterize the types and frequency of occurrence of MTPs and identify the most common medication classes associated with MTPs in participants enrolled in K-CHAMP who had at least 1 medication review completed. In addition, the average number of MTPs per participant was assessed in participants with any MTP at baseline who had at least 1 follow-up medication review encounter. The cumulative rate of resolution of MTPs from baseline to 12-month follow-up was assessed. As changes to clinical practice guidelines for CKD management during the later part of our study period strongly influenced the indication without drug therapy category (ie, expanded indications for SGLT-2 inhibitors) only MTPs present at baseline were included to assess longitudinal effects of the K-CHAMP intervention on MTPs.

### Statistical Analysis

Means with standard deviations and counts with percentages were used to descriptively summarize participant characteristics. To characterize the rate of MTPs over time, we calculated the ratio of the total MTPs observed to the number of participants that contributed to the count at baseline, 6 months, and 12 months across all and stratified based on MTP category. We examined the association between the mean number of MTPs and baseline participant characteristics derived from EHR (age, legal sex (female/male), race, CKD stage, rural-urban commuting area, and polypharmacy) using the *t* test and reported the estimated means along with associated 95% confidence intervals. Among existing MTPs at baseline, we determined the ones that were resolved at subsequent timepoints. The resolution endpoint was considered met if the category- and drug-specific MTP was no longer present, as ascertained during follow-up medication management encounters. To estimate the cumulative probability of resolution over time, we performed discrete-time survival analyses using a generalized linear mixed model with binomial link and complementary log-log link, which affords a proportional hazards interpretation. The model included practice-level random effects to account for clustering within practices. Participants who dropped out, did not have follow-up medication management encounter, or reached a trial endpoint (≥40% eGFR decline, kidney failure, death, or medical management without dialysis) were censored. The hazards of resolution were estimated at each timepoint using the marginal effects package and were converted to cumulative probabilities using the product-limit method.^30^ Point estimates and 95% confidence intervals were reported. *P* values < 0.05 were considered statistically significant. All analyses were performed using R version 4.3.1 (R Core Team).^31^

## Results

Among the 754 participants enrolled in the intervention arm between May 2019 and November 2021, baseline medication review was completed for 730 (97%) participants, and the remaining 24 (3%) participants opted out of medication review. Among the 730 participants, baseline medication review was completed via telephone for 381 (52%) and by chart review only for 349 (48%). Over a median follow-up period of 17 ± 8 months, a total of 1,503 medication reviews were completed, corresponding to an average 2 medication review encounters per participant. Among participants who did not meet a trial endpoint, medication review was completed for 587 of 702 participants (84%) at 6-month follow-up, and for 339 of 562 (60%) participants at 12-month follow-up, either via telephone or via chart review (Table S1).

Baseline characteristics are summarized in Table 2. The average age was 74 ± 9 years and eGFR was 37 ± 8 mL/min/1.73 m^2^. Most participants were White (93%) and women (55%). Common comorbid conditions included hypertension (94%), cardiovascular disease (79%), and diabetes (64%). Participants took an average of 6 prescribed medications, and polypharmacy was observed in 60%. Participants receiving telephonic medication review at baseline were more likely metropolitan living compared with those who received chart review alone. Those who received telephonic medication review had lower diastolic blood pressure at baseline compared with those who received chart review alone (73 mm Hg vs 75 mm Hg, respectively); however, this difference is not considered clinically significant.

**Table 2:** Baseline Characteristics

Variable	Overall N = 730 Mean (SD) or N (%)	Chart Review N = 349 Mean (SD) or N (%)	Phone Review N = 381 Mean (SD) or N (%)	*P* Value
Age (y)	74 (9)	73 (9)	74 (9)	0.3
Female sex	398 (55)	202 (58)	196 (51)	0.08
Race				0.5
African American	48 (7)	27 (8)	21 (6)	
White	675 (93)	3 (1)	4 (1)	
Others	7 (1)	319 (91)	356 (93)	
RUCA				0.01
Metropolitan	548 (75)	247 (71)	301 (79)	
Rural/micropolitan	181 (25)	101 (29)	80 (21)	
CHF	242 (33)	116 (33)	126 (33)	>0.9
DM				0.6
Type 1	14 (2)	5 (1)	9 (2)	
Type 2	453 (62)	219 (63)	234 (61)	
HTN	688 (94)	331 (95)	357 (94)	0.5
CVD	575 (79)	274 (79)	301 (79)	0.9
Charlson comorbidity index	6.8 (2.8)	6.8 (2.8)	6.9 (2.8)	0.7
BMI (kg/m^2^)	32.4 (7.6)	32.7 (7.8)	32.1 (7.4)	0.3
Systolic BP (mm Hg)	131 (16)	132 (16)	130 (16)	0.2
Diastolic BP (mm Hg)	74 (10)	75 (11)	73 (10)	<0.001
HbA1C (%)	7.0 (1.5)	7.0 (1.5)	7.0 (1.4)	0.7
Cr (mg/dL)	1.7 (0.4)	1.7 (0.4)	1.7 (0.4)	0.2
eGFR (mL/min/1.73 m^2^)	37 (8)	37 (8)	37 (8)	0.5
UACR (mg/g), median (IQR)	86 (16, 435)	89 (17, 472)	79 (16, 414)	0.4
KFRE 5-y risk, % median (IQR)	4.2 (2.3, 9.1)	4.3 (2.3, 9.5)	4.1 (2.3, 8.9)	0.6
CKD stage				>0.9
2	6 (1)	2 (1)	4 (1)	
3a	96 (13)	46 (13)	50 (13)	
3b	509 (70)	242 (69)	267 (70)	
4	119 (16)	59 (17)	60 (16)	
Albuminuria stage				0.9
A1	227 (34)	106 (33)	121 (34)	
A2	228 (34)	106 (33)	122 (34)	
A3	220 (33)	107 (34)	113 (32)	
No. of prescribed medications	6 (4)	6 (4)	6 (4)	0.7
Polypharmacy (≥5 prescribed medications)	441 (60)	218 (62)	223 (59)	0.3
Hyperpolypharmacy (≥10 prescribed medications)	106 (15)	56 (16)	50 (13)	0.3
NSAID	43 (6)	19 (5)	24 (6)	0.6
ACEi/ARB	333 (46)	159 (46)	174 (46)	>0.9
SGLT-2i	22 (3)	13 (4)	9 (2)	0.3
GLP-1RA	47 (6)	24 (7)	23 (6)	0.6
Statin (moderate or high intensity)	368 (50)	176 (50)	192 (50)	>0.9

Abbreviations: ACEi, angiotensin-converting enzyme inhibitor; ARB, angiotensin II receptor blocker; BMI, body mass index; BP, blood pressure; CHF, congestive heart failure; CKD, chronic kidney disease; Cr, serum creatinine; CVD, cardiovascular disease; DM, diabetes mellitus; eGFR, estimated glomerular filtration rate; GLP-1RA, glucagon-like peptide-1 receptor agonist; HbA1c, hemoglobin A1c; HTN, hypertension; IQR, interquartile range; KFRE, kidney failure risk equation; NSAID, nonsteroidal anti-inflammatory drug; RUCA, rural-urban commuting area ; SGLT-2i, sodium-glucose cotransporter-2 inhibitor; UACR, urine albumin-to-creatinine ratio.

### Baseline MTPs and Medication Discrepancies

**Figure 1:**
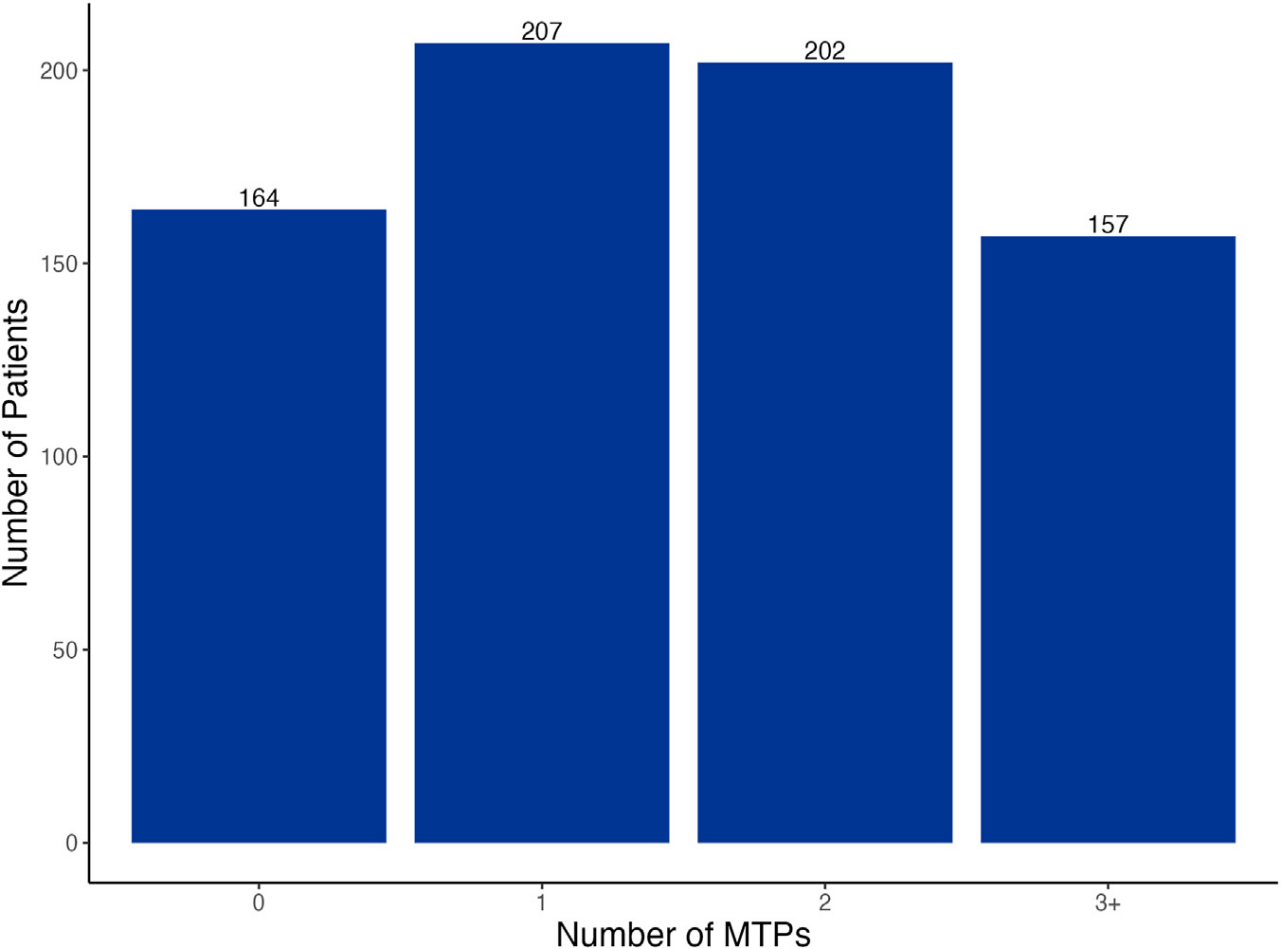
Distribution of MTPs at baseline. MTP, medication therapy problem.

**Figure 2:**
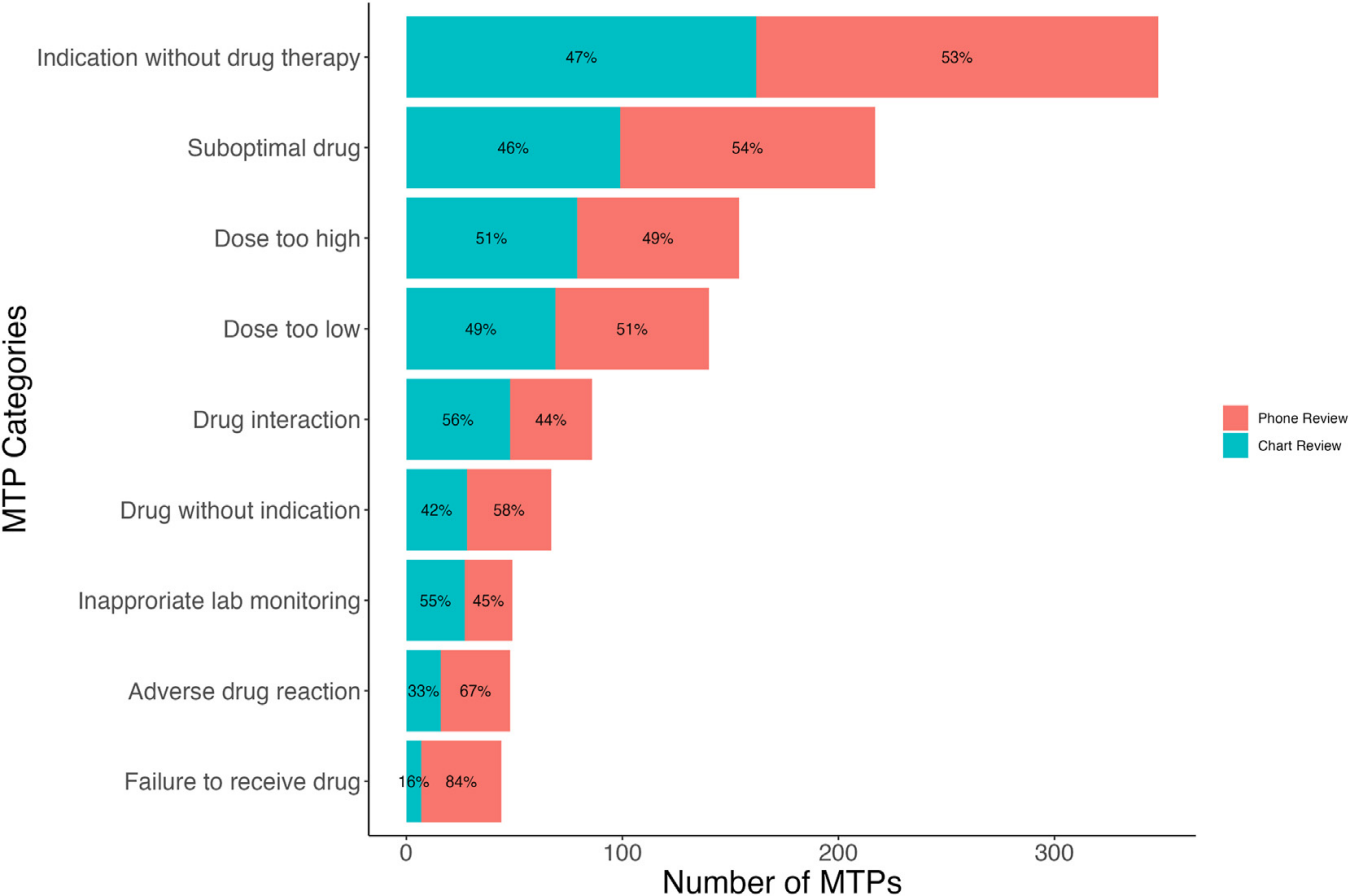
Number of MTPs at baseline based on MTP category. MTP, medication therapy problem.

**Figure 3:**
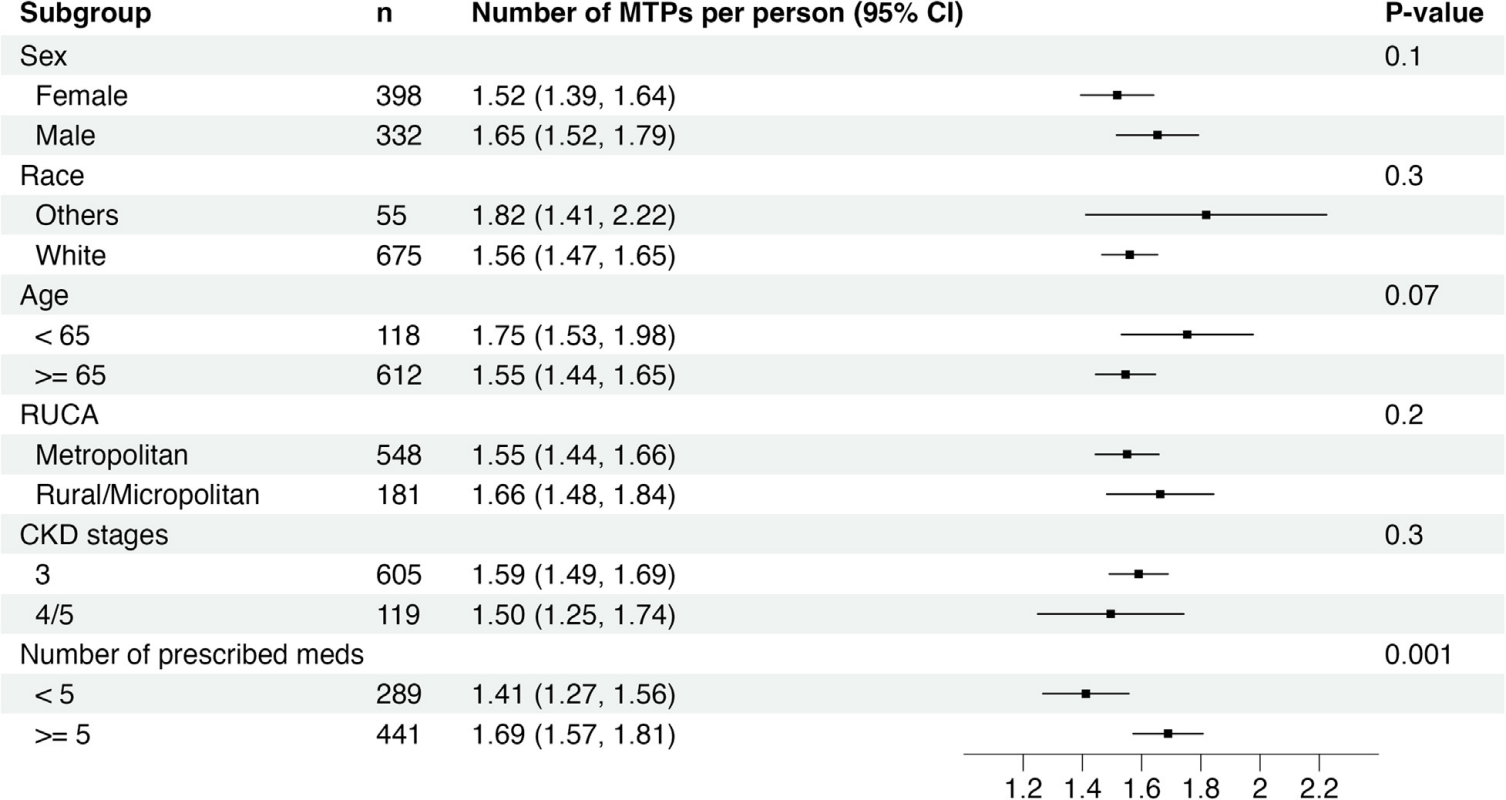
Mean number of MTPs at baseline according to subgroups. CI, confidence interval; CKD, chronic kidney disease; MTP, medication therapy problem; RUCA, rural-urban commuting area.

A total of 1,153 MTPs were identified in 566 participants (78%) at baseline, with 207 (28%), 202 (28%), and 157 (22%) participants experiencing 1, 2, and 3 or more MTPs, respectively (Fig 1). The most common MTPs at baseline were indication without drug therapy, suboptimal drug, and dosage too high (Fig 2). Drug use without indication, adverse drug reaction, and failure to receive drug were more often identified in patients who completed baseline CMR by phone. SGLT-2 inhibitors were the most frequent medication class implicated for indication without drug therapy (33%; Table 1). Nonsteroidal anti-inflammatory drugs were the most common suboptimal medications (28%), and metformin was the most common medication dosed too high (24%). In subgroup analysis, polypharmacy was associated with a higher mean number of MTPs per participant (1.69 vs 1.41; *P* = 0.001) compared with participants without polypharmacy. There was no significant difference in MTPs per participant across subgroups of sex, race, age, CKD stage, or rural/urban commuting area (Fig 3).

**Figure 4:**
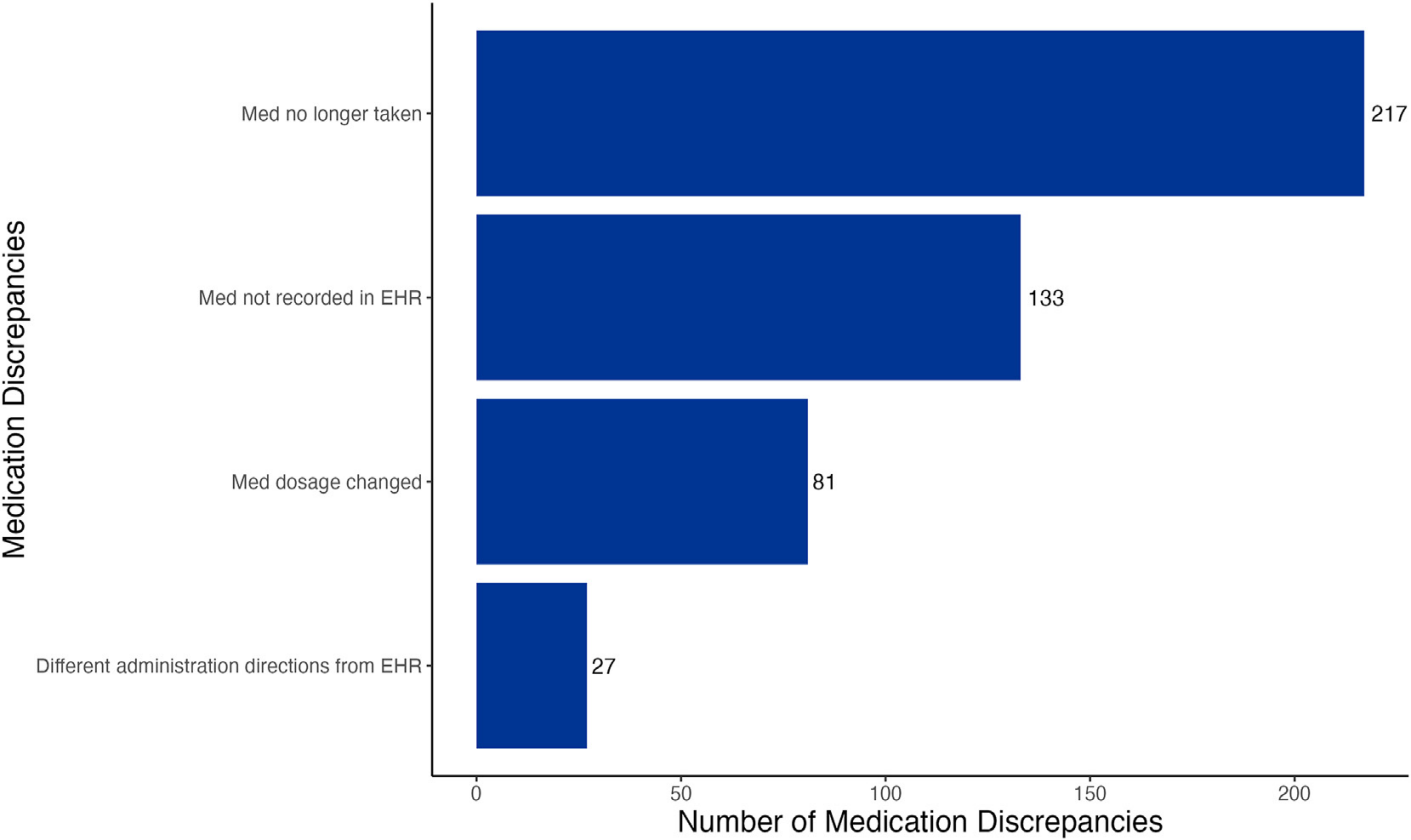
Medication discrepancies at baseline in participants completing CMR by phone. CMR, comprehensive medication review; EHR, electronic health record.

In participants completing baseline CMR by telephone, 302 participants (79%) had a medication discrepancy at baseline, such as drug no longer taken (47%), drug not recorded (29%), drug dose changed (18%), and different administration directions (6%; Fig 4).

### MTP Resolution During K-CHAMP Intervention

**Figure 5:**
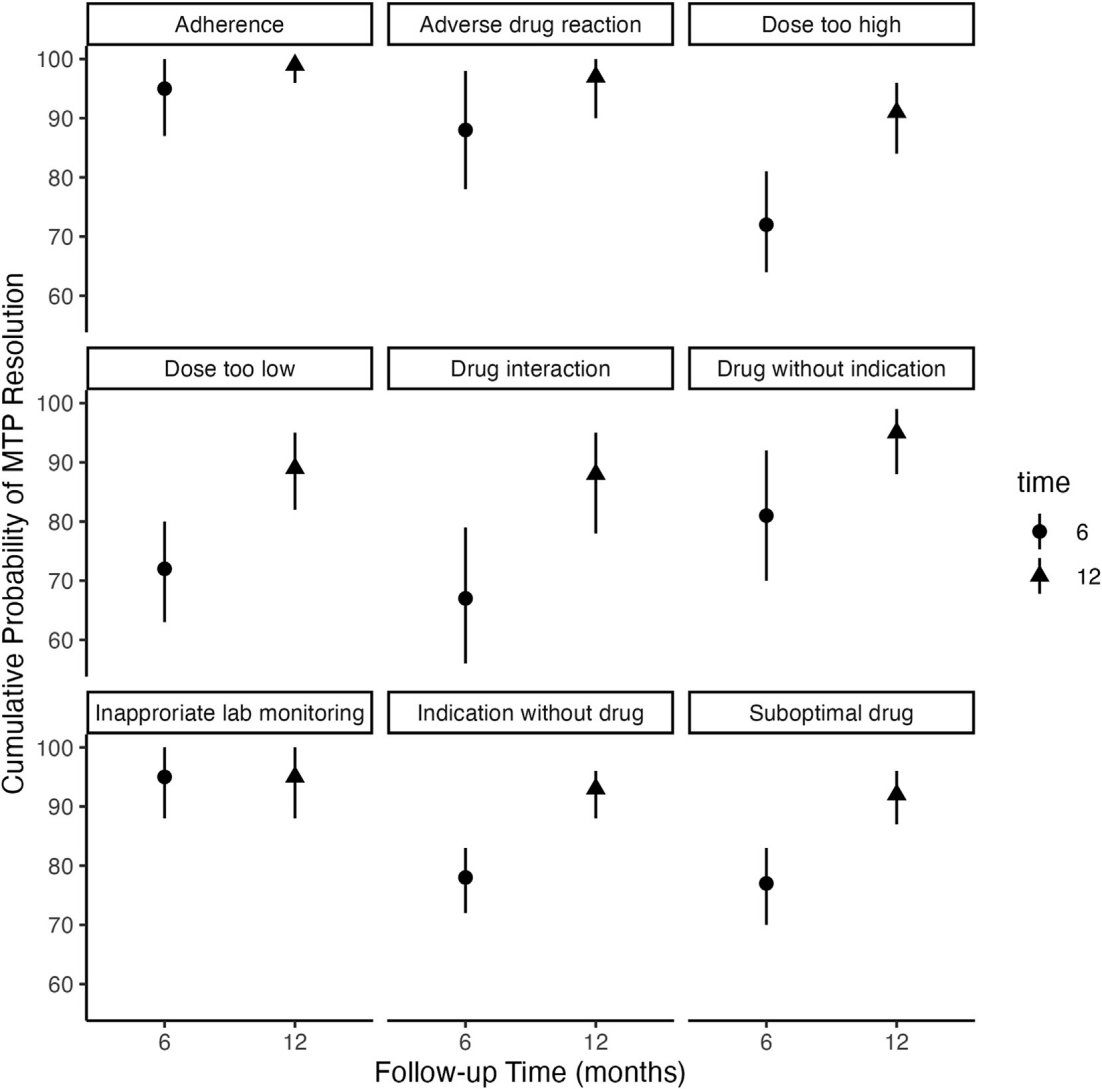
Cumulative probability of MTP resolution at 6-month and 12-month follow-up based on MTP category. MTP, medication therapy problem.

Of the 566 participants with MTP at baseline, 454 participants (80%) had at least 1 follow-up medication review (Table 3). Staffing shortages during the coronavirus disease-2019 pandemic contributed to only 60% of participants receiving medication review at 12-month follow-up. Thus, we used mixed-methods modeling to estimate MTP resolution rate. The average number of MTPs per participant decreased from 2.01 at baseline to 1.28 at 6 months (36% reduction), and 1.15 at 12 months (43% reduction). Based on the discrete-time survival model, 92% (95% confidence interval, 90-95) of MTPs were resolved by 12 months, with 77% resolving within 6 months and 15% between 6 and 12 months. The largest change in resolution rate was in the drug interaction MTP category (Fig 5).

**Table 3:** MTPs at Baseline and Follow-up in Participants With At Least One Follow-up Medication Review Completed

MTP Categories	Baseline			Follow-up 6 Mo			Follow-up 12 Mo		
	No. of MTP	Participants With MTP	MTP Per Person^a^	No. of MTP	Participants With MTP	MTP Per Person^a^	No. of MTP	Participants With MTP	MTP Per Person^a^
Indication without drug therapy	277	240	0.61	66	62	0.40	8	7	0.24
Suboptimal drug	179	154	0.39	43	41	0.26	6	6	0.18
Dosage too high	124	110	0.27	31	30	0.19	9	9	0.26
Dosage too low	115	110	0.25	33	33	0.20	9	9	0.26
Drug use without indication	48	45	0.11	9	9	0.05	1	1	0.03
Drug interaction	67	55	0.15	21	20	0.13	5	5	0.15
Inappropriate laboratory monitoring	38	34	0.08	2	2	0.01	0	0	0
Adverse drug reaction	39	33	0.09	5	4	0.03	1	1	0.03
Failure to receive drug	30	22	0.07	2	2	0.01	0	0	0
All MTPs	917	454	2.02	212	166	1.28	39	34	1.15

Abbreviation: MTP, medication therapy problem.

a MTP per person is per total number of participants with MTP at each time period.

## Discussion

This study demonstrates high medication burden and prevalence of MTPs in individuals with moderate- to high-risk CKD in a primary care setting. We identified at least 1 MTP in 78% of participants at baseline. We demonstrate that a population health management approach with pharmacist-led medication review is highly effective at resolving MTPs and increasing the use of medications proven to reduce CKD progression.

Our study included people with CKD with moderate to high risk of progression who were not receiving outpatient nephrology care. Most care for people with nondialysis-dependent CKD is provided by PCPs—the United States Renal Data System reports that only 26%, 53%, and 42% of Medicare beneficiaries with CKD G3, G4, and G5, respectively, had a nephrology physician outpatient encounter in 2021.^32^ Thus, crucial elements of CKD care, including optimizing medication regimens, are needed in the primary care setting. Although several studies have characterized MTPs in people with dialysis-dependent CKD,^2^^,^^10^^,^^11^ or in hospitalized patients with CKD,^12^ studies reporting MTPs in the ambulatory, nondialysis-dependent CKD population are fewer. Our study fills this knowledge gap by providing a detailed characterization of MTPs in people with CKD across a large health system, enrolling 25% of participants from rural and/or micropolitan areas where access to specialty care may be limited. In our study, the most commonly identified MTP was indication without drug, inferring the need for additional drug therapy. This is not surprising, given evidence to support the expanded use of SGLT-2 inhibitors and glucagon-like peptide-1 receptor agonists in people with CKD throughout our study period.^25^ Of concern, however, is the need for additional medication therapy in patients who are already burdened with polypharmacy. In our study, 60% of participants had polypharmacy at baseline, and it was significantly associated with a higher number of MTPs per participant in subgroup analysis. Although polypharmacy is associated with poor outcomes,^4^ medications with appropriate indications should not be withheld in an effort to reduce medication burden. Health care teams should be attentive to addressing inappropriate polypharmacy in people with CKD, which may include a medication review process to evaluate medication indication and discuss deprescribing.^6^ Another important finding of our study was a high prevalence of medication discrepancies, similar to the frequent medication discrepancies reported in individuals with late-stage CKD and kidney failure.^33^^,^^34^ This highlights the inaccuracy of EHR medication lists and the need for regular review of patient-reported medications. Medications not recorded in the EHR accounted for 29% of all medication discrepancies in our study, raising concern for underreported over-the-counter nonsteroidal anti-inflammatory drug or nephrotoxic herbal supplement use in people with CKD.

Only a handful of interventions targeting medication optimization in nondialysis CKD have been rigorously tested. Most aimed to reduce medication errors, and very few have targeted overall medication optimization including guideline-concordant medication uptake. Tuttle et al^13^ tested the effect of a pharmacist-led Medication Therapy Management intervention on 90-day acute health care use in 141 participants with nondialysis-dependent CKD post hospital discharge. In this study, 92% of participants had an MTP, of which 71% were resolved during the pharmacist encounter, but the intervention did not reduce acute care use or improve guideline-concordant CKD care. The higher MTP rate in this study is reflective of medication changes post hospitalization, and perhaps differences in definitions of medication problems used in our study. Two studies have evaluated MTPs in people with CKD in community pharmacies.^35^^,^^36^ Joosten et al assessed medication errors in 1,368 individuals with an eGFR of ≤40 receiving care from 11 primary care pharmacies; pharmacists recognized medication errors in 15% of participants and prescribers accepted 66% of medication adjustment recommendations.^36^ Lalonde et al report that community pharmacists enrolled in a training-and-communication network program (ProFiL) identified on an average 2.16 MTPs per participant among 442 individuals with CKD, and the mean number of MTPs per participant decreased by 15% in the intervention group after 12 months.^35^ In our study, MTPs per participant decreased by 43% at 12 months. We speculate that the higher per-person MTP resolution rate in our study compared to prior studies is due to more comprehensive management that included additional PCP guidance through e-consult and ongoing academic detailing, EHR integration, and PCP workflow alignment, and this made our intervention more impactful.

Our study builds on these prior studies and provides evidence in the under-studied ambulatory CKD population on the important value of clinical pharmacists in identifying and resolving MTPs. An additional unique strength of our study is the focus of the K-CHAMP intervention on the optimization of guideline-concordant medication use in CKD. In addition to recognizing medication discrepancies and MTPs related to safety, we identified and characterized MTPs related to indication (indication without drug therapy and drug use without indication) and effectiveness (dose too low), seeking improvement in guideline-concordant medication uptake in our population. In a study of 256 CKD clinic patients at the transition of care between nephrologists and PCPs, Schutze et al assessed the effect of adding a pharmacist to the clinical team on the prevalence of MTP and PCP acceptance of nephrology recommendations.^37^ Similar to our study, Schutze et al^37^ found that “(clear) indication, but no drug prescribed,” was the most common MTP category, and MTPs in this category were significantly reduced in the intervention group compared with control at the transition from nephrologist to general practitioner care and at 6-month follow-up. These studies suggest that leveraging multidisciplinary teams, including pharmacists, to implement comprehensive care for people with CKD can identify and minimize gaps in guideline-concordant CKD care.

## Limitations

Several important limitations of our study should be considered. First, medication management was not done for our usual-care control-arm participants, as it would have been unethical to identify MTPs without intervening. Thus, how the MTP resolution rate in K-CHAMP compares with usual care is unknown. However, for our most common MTP—indication without drug therapy—which was mainly driven by the new cardio-kidney protective medications, we previously showed that intervention significantly increased the use as compared with controls.^17^ MTPs related to medication adherence may have been underreported in our study as medication adherence was only assessed for participants who were able to be contacted. In addition, no standardized tool was used to assess medication adherence. Similarly, reliance on chart review for participants who were unable to be reached by phone may have affected MTP identification owing to unknown accuracy of the EHR-reported medication list and limited ability to assess over-the-counter medication use, medication side effects, and adherence by chart review alone. Of note, our contact rate for telephonic CMR (∼50% at baseline) is comparable with or better than other similar programs. For example, of patients eligible for CMR under the Medicare Part D Enhanced Medication Therapy Management Model (∼250,000 patients per year during 2017-2021), only about a third of beneficiaries received the service.^26^ Notably, the most common MTP identified in our population, indication without drug therapy, was unlikely to be affected substantially by modality of medication review. The most common medications associated with this MTP category (SGLT-2 inhibitor, angiotensin-converting enzyme inhibitor/angiotensin II receptor blocker, statin) are likely to be prescribed by PCP or specialty providers (ie, cardiology and endocrinology) in the University of Pittsburgh Medical Center Health System and thus documented on the EHR medication list and/or dispense report history.

Our population had rural-urban diversity, but lacked racial and ethnic diversity, which is reflective of Western Pennsylvanian communities^38^; this may limit the generalizability of our findings. Our median 17-month follow-up limits the ability to observe long-term improvements in MTP reduction. Last, we were unable to evaluate the effect of individual intervention components as this was part of a multicomponent intervention. However, such multidisciplinary team care is needed and advocated to optimize CKD care.

In conclusion, our study demonstrates that individuals with nondialysis-dependent CKD have a high prevalence of MTPs. Multidisciplinary team care may facilitate identification, resolution, and prevention of MTPs, and promote implementation of guideline-concordant care in people with CKD. Our novel population health management--based approach that offered multidisciplinary team care including pharmacist expertise to comanage patients with PCPs within a large health system across Western Pennsylvania is a potential scalable strategy to improve CKD outcomes and medication safety.

## Supplementary Materials

Supplementary File (PDF) Box S1; Table S1.
