# Supplementary material for: Effect of a Population Health Management Intervention on Medication Therapy Problems in People With Chronic Kidney Disease: Post Hoc Analysis of the K-CHAMP Cluster-Randomized Trial
Source: Kidney Med. 2025 Mar 18;7(5):100995. doi: 10.1016/j.xkme.2025.100995 (PMC12051626; doi:10.1016/j.xkme.2025.100995)
Supplement: Supplementary File (PDF) — Box S1; Table S1. [file mmc1.pdf]

Box S1.

Kidney CHAMP

A medication reconciliation and safety review was conducted for @NAME@ on @TODAYDATE@ as part of Kidney CHAMP.
Recommendations are listed below. Additional recommendations may be provided by the nephrologist in the e-consult.

PharmD name
Phone #

Recommendations

- Case reviewed with nephrology e-consult team. Please see medication-related recommendations in the nephrology e-consult.
- Additionally:
    -

Assessment

- Medication Discrepancies/Issues Reported:
    -
- Recent CKD-related labs:
    - Scr =
    - eGFR =
    - K =
    - Weight =
    - UACR =
- Hypertension
    - Regimen includes:
    - Home BP readings:
    - BP in office:
    - Weight/fluid/edema:
    - Recommendations:
- Diabetes
    - Regimen includes:
    - Most recent A1c:
    - Patient checks FBG at home and reports ranges of: .
    - No reports of hypoglycemia. Able to recognize symptoms if hypoglycemia occurs.
    - Patient not checking FBG at home.
    - Recommendations:
- Hyperlipidemia
    - Current regimen:
    - ASCVD risk:
    - Lipid panel:
    - Recommendations:
- Vaccinations
    - Received:
    - Recommend:
- OTC/NSAIDs:
- Other Recommendations:
- Drug-Drug Interactions:

Updated patient reported medication list:
@MEDC2@

Disclaimer: These recommendations are solely based on patient reported medication list/interview and review of the patient information in the chart. The Primary Care Provider is responsible for the final decision on any recommendations.

Table S1. Medication reviews by modality at 6- and 12-months follow up

	**6 Month follow-up (n=586)**		**12 Month follow follow-up (n=339)**	
**Baseline**	Chart Review	Phone Review	Chart Review	Phone Review
**Chart Review**	195	71	102	38
**Phone Review**	173	147	103	96
